# Draft genome sequence of *Staphylococcus aureus* N208 isolated from a nasal swab of a healthy female

**DOI:** 10.1128/mra.00811-25

**Published:** 2025-09-12

**Authors:** Sandra Jablonska, Lila Nelson, Grace Finger, Alex Kula, Catherine Putonti

**Affiliations:** 1 Bioinformatics Program, Loyola University Chicago 2456, Chicago, Illinois, USA; 2 Biology Department, Loyola University Chicago 224010, Chicago, Illinois, USA; Wellesley College, Wellesley, Massachusetts, USA

**Keywords:** *Staphylococcus aureus*, nasal microbiome, methicillin susceptible *S. aureus*, MSSA

## Abstract

*Staphylococcus aureus*, a common member of the healthy human microbiota, is an opportunistic pathogen. To investigate the genomic features of this species in healthy individuals, we present the draft genome sequence of *S. aureus* N208, isolated from a nasal swab collected from a healthy female participant.

## ANNOUNCEMENT

*Staphylococcus aureus* is an opportunistic pathogen, colonizing the anterior nares as a commensal member of approximately 20%–30% of healthy adults without causing disease (1). Nevertheless, carriage of *S. aureus* has the potential for transmission, thus necessitating monitoring of antibiotic resistance within strains circulating among healthy individuals (2). In this study, we present a draft genome assembly of *S. aureus* N208 isolated from a nasal swab of a healthy female.

Participants in our institutional review board (IRB)-approved study (IRB no. 3603) self-identified as female, “healthy,” and had not taken antibiotics within the last 6 months. Participants were students at Loyola University Chicago (Chicago, IL). Each participant was instructed to rub the cotton swab (BD BBL CultureSwab) on the lower inside of the nostril for 30 s, after which the swab was inserted into the provided storage solution (Amies medium). Within 1 h of sampling, swabs were swirled and squeezed in the storage medium and then spread on mannitol salt (MS) agar plates, a selective medium for staphylococci. Plates were incubated for 24 h at 35°C with 5% CO_2_. Morphologically distinct colonies were purified via successive rounds of MS liquid culture and plating. DNA was extracted using the DNeasy Blood and Tissue kit (Qiagen), following the manufacturer’s protocol for Gram-positive bacteria. DNA was sent to SeqCoast (Portsmouth, NH USA) who performed library construction using the Illumina DNA Prep tagmentation kit and unique dual indexes and sequencing on the Illumina NextSeq2000 platform using a 300-cycle flow cell kit (2 × 150 bp reads). SeqCoast performed read demultiplexing, read trimming, and run analytics using DRAGEN v3.10.12 (Illumina) prior to delivery.

We used BV-BRC (v3.51.7) to assemble the genome specifying the “auto” option (3). First, BV-BRC trimmed the raw reads (trim_galore v0.6.5dev; https://github.com/FelixKrueger/TrimGalore) and then normalized the trimmed reads (bbnorm v37.60 https://sourceforge.net/projects/bbmap/). These reads were then assembled by Unicycler (v0.4.8) (4), and the assembly was polished (two rounds) by Pilon v1.24 (5), and genome statistics were generated. Taxonomic identification was confirmed by JSpeciesWS (6), determining an ANI value of 98.82% to *S. aureus* 502A (GCF_000597965.1), well above the threshold for species classification. Genome annotations were generated using the NCBI Prokaryotic Genome Annotation Pipeline (PGAP) v6.10 (7), including antibiotic resistance genes via AMRFinderPlus (8). Completeness and contamination metrics were computed using CheckM v1.2.3 (9) upon submission to NCBI. Unless otherwise noted, default parameters were used for all software tools.

Table 1 lists the assembly statistics for the *S. aureus* N208 genome. While the genome encodes for *blaZ*, which is common among *S. aureus* (see review [10]), it does not encode for *mecA*. Thus, the strain is predicted to be methicillin-susceptible.

**TABLE 1:** Genome assembly statistics for *S. aureus* N208

	*S. aureus* N208
Assembly length (bp)	2,669,603
No. of contigs	20
Contigs N50 (bp)	436,906
Coverage (x)	258.32
G + C (%)	32.5
# genes	2,633
# tRNAs	54
Completeness (%)	98.37
Contamination (%)	0.39
No. of reads	6,032,836
SRA accession no.	SRR34298193
Assembly accession no.	JBPFAL000000000

## Data Availability

The raw reads are publicly available through the SRA database (SRR34298193). This Whole Genome Shotgun project has been deposited in DDBJ/ENA/GenBank under the accession no. JBPFAL000000000. The version described in this paper is the first version, JBPFAL01000000.
